# Dynamic karyotype evolution and unique sex determination systems in *Leptidea* wood white butterflies

**DOI:** 10.1186/s12862-015-0375-4

**Published:** 2015-05-19

**Authors:** Jindra Šíchová, Anna Voleníková, Vlad Dincă, Petr Nguyen, Roger Vila, Ken Sahara, František Marec

**Affiliations:** Institute of Entomology, Biology Centre CAS, 370 05 České Budějovice, Czech Republic; Faculty of Science, University of South Bohemia, 370 05 České Budějovice, Czech Republic; Biodiversity Institute of Ontario, University of Guelph, N1G 2W1 Guelph, ON Canada; Institut de Biologia Evolutiva (CSIC-Universitat Pompeu-Fabra), 08003 Barcelona, Spain; Laboratory of Applied Entomology, Faculty of Agriculture, Iwate University, Morioka, 020-8550 Japan

**Keywords:** Lepidoptera, Wood white butterflies, Karyotype variability, Fluorescence *in situ* hybridization Chromosome fusion and fission, Multiple sex chromosomes, Speciation

## Abstract

**Background:**

Chromosomal rearrangements have the potential to limit the rate and pattern of gene flow within and between species and thus play a direct role in promoting and maintaining speciation. Wood white butterflies of the genus *Leptidea* are excellent models to study the role of chromosome rearrangements in speciation because they show karyotype variability not only among but also within species. In this work, we investigated genome architecture of three cryptic *Leptidea* species (*L. juvernica*, *L. sinapis* and *L. reali*) by standard and molecular cytogenetic techniques in order to reveal causes of the karyotype variability.

**Results:**

Chromosome numbers ranged from 2n = 85 to 91 in *L. juvernica* and 2n = 69 to 73 in *L. sinapis* (both from Czech populations) to 2n = 51 to 55 in *L. reali* (Spanish population). We observed significant differences in chromosome numbers and localization of cytogenetic markers (rDNA and H3 histone genes) within the offspring of individual females. Using FISH with the (TTAGG)_*n*_ telomeric probe we also documented the presence of multiple chromosome fusions and/or fissions and other complex rearrangements. Thus, the intraspecific karyotype variability is likely due to irregular chromosome segregation of multivalent meiotic configurations. The analysis of female meiotic chromosomes by GISH and CGH revealed multiple sex chromosomes: W_1_W_2_W_3_Z_1_Z_2_Z_3_Z_4_ in *L. juvernica*, W_1_W_2_W_3_Z_1_Z_2_Z_3_ in *L. sinapis* and W_1_W_2_W_3_W_4_Z_1_Z_2_Z_3_Z_4_ in *L. reali*.

**Conclusions:**

Our results suggest a dynamic karyotype evolution and point to the role of chromosomal rearrangements in the speciation of *Leptidea* butterflies. Moreover, our study revealed a curious sex determination system with 3–4 W and 3–4 Z chromosomes, which is unique in the Lepidoptera and which could also have played a role in the speciation process of the three *Leptidea* species.

**Electronic supplementary material:**

The online version of this article (doi:10.1186/s12862-015-0375-4) contains supplementary material, which is available to authorized users.

## Background

Speciation, *i.e.* the origin of new species, is a complex evolutionary process which leads to the formation of barriers preventing gene flow between emerging species. Defining the factors that generate such barriers is a central goal for evolutionary biologists. Among animals, moths and butterflies (insect order Lepidoptera) represent an ideal model group for the study of various aspects of speciation. This is mainly due to the immense diversity of Lepidoptera, which include nearly 160,000 species and belong to the most speciose groups of animals [1]. Moreover, the study of moths and butterflies provides a number of practical advantages. Many species can be easily collected in the field, reared and hybridized in laboratory conditions and experiments can be replicated fairly often due to the relatively short generation time of many species.

Among traditional models, the *Heliconius* butterflies have been the subject of a high number of evolutionary studies showing that various wing patterns, resulting from predator-induced selection through Müllerian mimicry, ultimately lead to divergence and speciation (*e.g.* [2–6]). The shift in colour pattern mimicry also played a key role in generating pre-mating isolation as male mate preferences often led to strong assortative mating between individuals with similar wing pattern phenotypes [7]. Swallowtail butterflies of the genus *Papilio* are another diverse group of Lepidoptera where the evolution of mimicry greatly contributed to their spectacular radiation [8–10]. Lepidoptera also include models for research of sex pheromone communication and its role as a pre-zygotic barrier [11, 12]. A well-known example is the European corn borer, *Ostrinia nubilalis*, which comprises two sympatric races that are prevented from mating by utilizing opposite sex pheromone isomers of the same compound [13, 14]. The butterfly subgenus *Agrodiaetus* is one of the few taxa where reinforcement of pre-zygotic isolation has been demonstrated [15].

It is generally accepted that chromosomal rearrangements have the potential to limit introgression and thus facilitate the development and maintenance of reproductive isolation by means of suppressed recombination [16–18]. Reduced recombination enables the accumulation of genetic incompatibilities and leads to divergence and speciation. Suppression of recombination is an intrinsic feature of sex chromosomes which were suggested to play a disproportionate role in lepidopteran speciation [19, 20]. Recent studies in geographic subspecies of wild silkworms, *Samia cynthia* ssp. (Saturniidae), suggest that chromosomal rearrangements resulting in multiple sex chromosomes may also contribute to the formation of reproductive barriers and thus promote divergence and eventually speciation [21, 22]. Moreover, the holokinetic nature of lepidopteran chromosomes, *i.e.* the lack of a distinct primary constriction (the centromere), is expected to facilitate karyotype evolution mainly via chromosomal fusion and fission by reducing the risk of formation of dicentric and acentric chromosomes [23]. However, results of comparative genomics revealed a high degree of conserved synteny of genes between the silkworm *Bombyx mori* (Bombycoidea) and several other lepidopteran species [24–30]. The extensive conservation of chromosome print across Lepidoptera suggests evolutionary stability of lepidopteran karyotypes, with most of haploid chromosome numbers ranging n = 28–32 [31] and the most common and probably also ancestral number of n = 31 [30, 32].

The remarkably stable chromosome numbers and highly conserved synteny of genes between chromosomes of distant species contrast with the exceptional diversity of karyotypes found in some lepidopteran taxa. Probably the greatest interspecific karyotype variation in the animal kingdom was found in blue butterflies (Lycaenidae: Polyommatinae) of the genus *Polyommatus* with haploid chromosome numbers ranging from n = 10 to n = 223 [15, 33–35]. The latter, observed in the Atlas blue, *Polyommatus atlantica*, represents the highest chromosome number not only of Lepidoptera but of all animals [36]. In addition, blue butterflies of the subgenus *Agrodiaetus* represent the group with the largest difference in the number of chromosomes between sister species. Karyotypes of *Polyommatus* (*Agrodiaetus*) *biruni* and *P.* (*A.*) *posthumus* consist of n = 10 and n = 90 elements, respectively, with no intermediate karyomorphs. The similarity in genome size of these closely related species suggests that the karyotype variation is not caused by polyploidy but arose through chromosomal rearrangements such as fusion and fission [37]. However, recent comparative phylogenetic studies found little evidence supporting the role of chromosomal rearrangements in the speciation of *Agrodiaetus* blues and rather stressed the importance of reinforcement of their pre-zygotic isolation [15].

Exceptional intraspecific variability of karyotypes was also found in wood white butterflies of the genus *Leptidea* comprising several Eurasian species [38, 39]. In this genus, chromosome numbers vary greatly between and within species. While two species with predominantly Eastern Palaearctic distribution, *L. morsei* and *L. amurensis*, probably have a constant number of chromosomes (n = 54 and n = 61, respectively; [40]), three cryptic species mainly from the Western Palaearctic have a variable number of chromosomes [38, 41]. The most striking case is *L. sinapis*, which shows a gradual decrease in the diploid chromosome number from 2n = 106 in Spain to 2n = 56 in eastern Kazakhstan, resulting in a 6000 km wide chromosomal cline of recent origin [39]. Excluding polyploidy, this is the widest known within-species chromosome number range for any animal or plant. Moreover, a variable number of chromosomes was described in the other two cryptic species, *L. reali* (2n = 52–54) and *L. juvernica* (2n = 80–84) [38].

Although the nature of dynamic evolution of *Leptidea* karyotypes and its role in speciation is not yet known, the chromosomal cline found in *L. sinapis* provided strong evidence for rapid and extensive within-species accumulation of numerous chromosomal rearrangements [39]. While such clinal speciation is theoretically possible, it is difficult to document without further research. In this study, we integrated standard cytogenetic techniques and FISH (fluorescence *in situ* hybridization) mapping of major ribosomal RNA (rRNA) and H3 histone genes to study among- and within-species variability in the karyotypes of three cryptic *Leptidea* species (*L. juvernica*, *L. sinapis* and *L. reali*). We also determined the sex chromosome constitution using genomic *in situ* hybridization (GISH) and examined molecular differentiation of the sex chromosomes through comparative genomic hybridization (CGH). Cytogenetic characteristics were compared with the aim of understanding karyotype and sex chromosome evolution in *Leptidea* butterflies.

## Results

### Molecular identification of *Leptidea* specimens

**Fig. 1:**
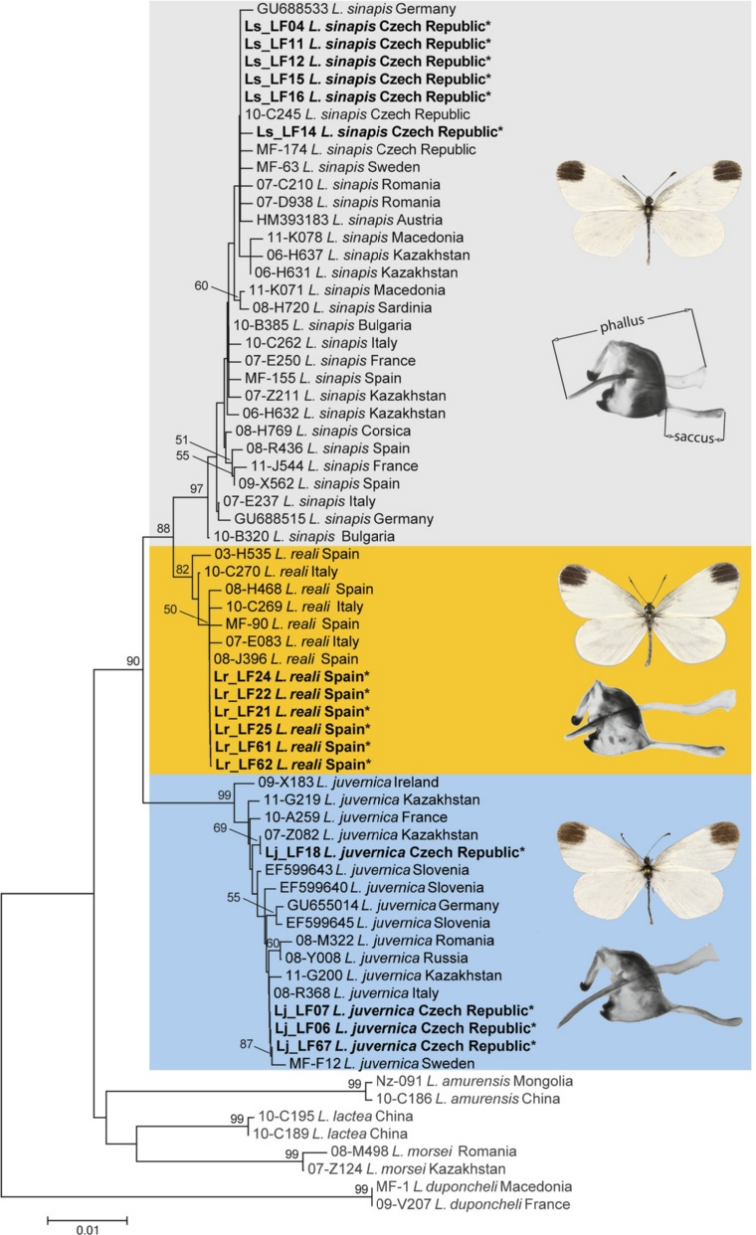
Neighbor-joining tree of mitochondrial *COI* haplotypes of *L. sinapis* (grey background), *L. reali* (orange background) and *L. juvernica* (blue background). Specimens sequenced and analysed in this study are indicated by an asterisk and were combined with representatives of all available haplotypes of *L. sinapis, L. reali* and *L. juvernica* identified in a previous study [41]. *Leptidea amurensis*, *L. lactea*, *L. morsei* and *L. duponcheli* were used as outgroup. For the origin of all specimens and GenBank accession numbers, see Additional file 5: Table S1. The scale represents 0.01 substitutions per site. Bootstrap supports (100 replicates) are shown next to the recovered nodes. Representative male specimens and genitalia (drawn to scale, with *phallus* and *saccus* indicated) are shown

Morphometric analysis of genitalia allowed us to identify only two groups, *L. sinapis* and the group consisting of *L. reali* and *L. juvernica*, whose genitalia cannot be reliably distinguished [38]. Phylogenetic analyses based on two DNA markers, the mitochondrial cytochrome *c* oxidase subunit 1 (*COI*) gene and the nuclear internal transcribed spacer 2 (*ITS2*) sequence, revealed three supported major clades corresponding to *L. juvernica*, *L. sinapis* and *L. reali* (Fig. 1 and Additional file 1: Figure S1). Relationships among these clades coincide with previous results [38, 41] with *L. juvernica* being sister to the species pair *L. sinapis* and *L. reali*.

### Karyotype differences in chromosome number and structure

Chromosome numbers of all three *Leptidea* species were counted from mitotic metaphase complements prepared from wing imaginal discs of the last instar larvae and stained by means of FISH with (TTAGG)_*n*_ telomeric probes (tel-FISH) to facilitate identification of individual chromosome elements. In each *Leptidea* species, several tens of mitotic metaphases were analysed in the progeny of individual females.

**Fig. 2:**
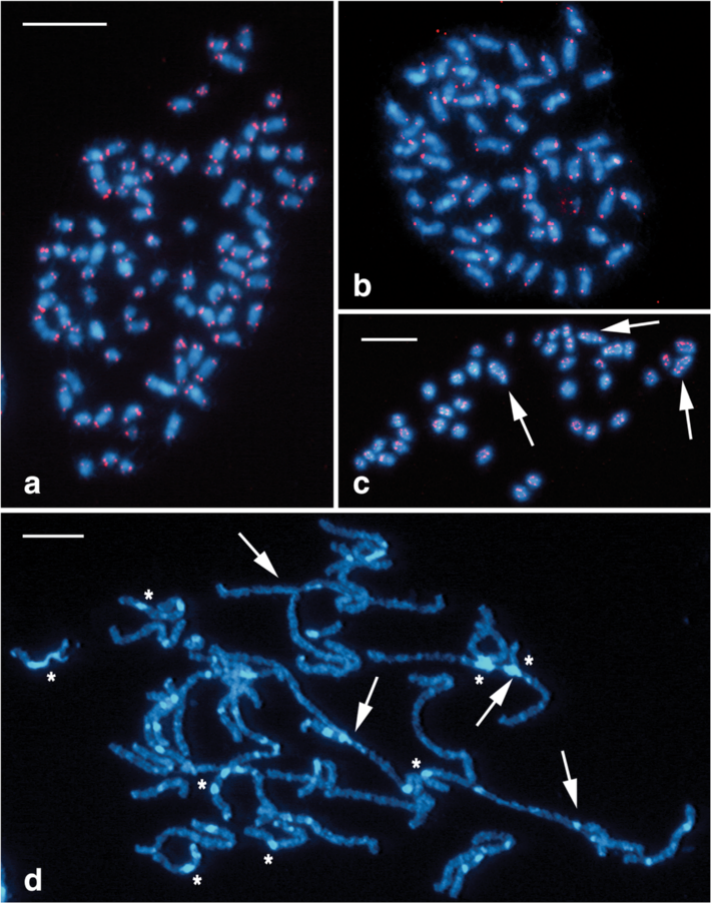
Karyotype analysis of mitotic and meiotic chromosomes of *Leptidea* species by FISH with the (TTAGG)_*n*_ telomeric probe. Hybridization signals of the Cy3-dUTP-labelled telomeric probe (red) indicate chromosome ends in (**a**–**c**). Chromosomes were counterstained with DAPI (blue). White arrows indicate chromosome multivalents and asterisks show heterochromatic blocks. **a** Mitotic metaphase of *L. juvernica* female with numerous middle- or small-sized chromosomes (2n = 85). **b** Mitotic metaphase of *L. reali* female with large chromosomes of a similar size (2n = 55). **c** Meiotic metaphase I of *L. juvernica* male showing several chromosome multivalents. **d** Meiotic pachytene complement of *L. juvernica* female showing several chromosome multivalents and numerous blocks of DAPI-highlighted heterochromatin. Scale bars = 10 μm; (**a**) and (**b**) have the same scale

**Fig. 3:**
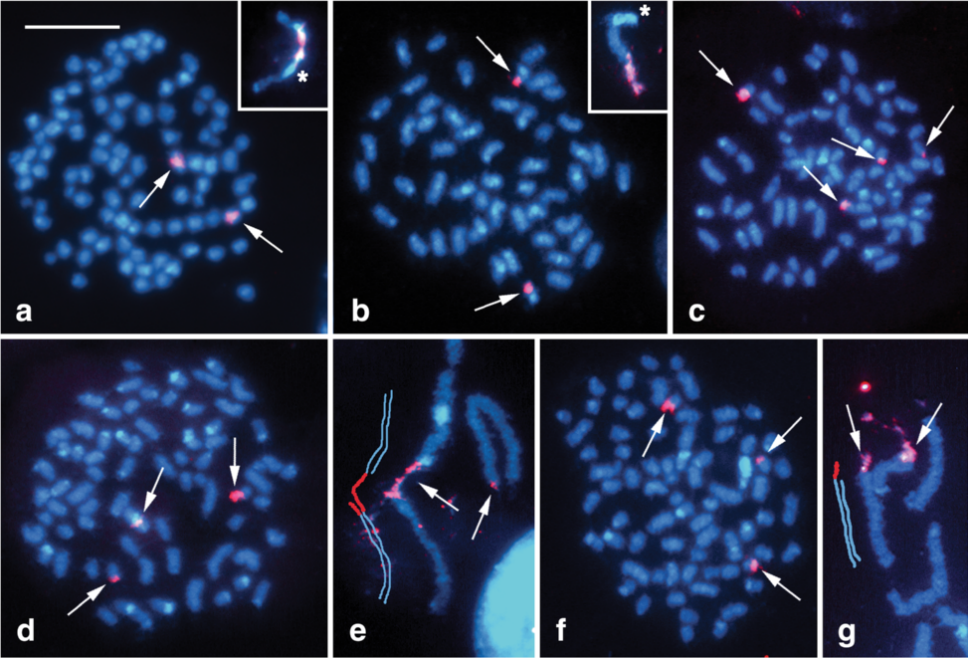
Localization of rDNA clusters in spread chromosome preparations of three *Leptidea* species by FISH with 18S rDNA probe. Chromosomes were counterstained with DAPI (blue). Arrows show hybridization signals of the 18S rDNA probe (red); asterisks indicate DAPI-positive blocks of heterochromatin. **a** Mitotic metaphase of *L. juvernica* male (2n = 90); the inset in the upper right corner shows the pachytene NOR-bivalent with a large interstitial rDNA cluster. **b** Mitotic metaphase of *L. reali* female (2n = 52); the inset in the upper right corner shows the pachytene NOR-bivalent with a large terminal rDNA cluster. **c** Male mitotic metaphase (2n = 69) with a typical hybridization pattern found in the offspring of one *L. sinapis* female. Figures (**d**–**g**) show a variable pattern in the offspring of another *L. sinapis* female: (**d**) mitotic metaphase of male offspring (2n = 71); (**e**) hybridization signals on pachytene chromosomes of the same male offspring (schematic drawing shows the structure of a trivalent carrying two out of three rDNA clusters); (**f**) mitotic metaphase of female offspring (2n = 73); (**g**) hybridization signals on pachytene chromosomes of the same female offspring (schematic drawing shows the structure of a bivalent heterozygous for a terminal rDNA cluster). Scale bar = 10 μm

Based on repeated counts we found that chromosome numbers differ considerably in all three species studied. Moreover, we observed differences in the number of chromosomes even among the offspring of individual females. We established that, in the population studied, the chromosome number is not fixed and ranges from 2n = 85 to 91 in *L. juvernica* and 2n = 69 to 73 in *L. sinapis* (both from Czech populations) to 2n = 51 to 55 in *L. reali* (Spanish population). Mitotic complements of *L. juvernica* and *L. sinapis* also displayed a higher variability in chromosome size, having mostly middle- or small-sized chromosomes (*L. juvernica*, Fig. 2a) or a mixture of large- and small-sized chromosomes (*L. sinapis*, Fig. 3c, d, f), while in *L. reali* we observed larger chromosomes of a similar size (Fig. 2b).

In male meiotic metaphase I (MI) and pachytene complements of all studied species we observed complex chromosomal rearrangements (Fig. 2c, d) and conspicuous heterochromatin blocks highlighted with DAPI (Fig. 2d). However, in female pachytene complements these DAPI-positive blocks did not allow the identification of a sex chromosome bivalent according to the W chromosome, which is usually the only largely heterochromatic element present in lepidopteran karyotypes [42, 43].

### Chromosomal location of major rDNA

FISH with the biotin-labelled 18S ribosomal DNA (rDNA) probe combined with the digoxigenin-labelled (TTAGG)_*n*_ telomeric probe did not reveal any difference in the number of major rDNA clusters in offspring of three *L. juvernica* and three *L. reali* females. In both species, the rDNA probe mapped to two mitotic metaphase chromosomes of a similar size (Fig. 3a, b; for simplification, hybridization signals of the telomeric probe are not shown) and to a single bivalent in the pachytene stage (insets of Fig. 3a, b). This clearly indicates the presence of a single pair of chromosomes, each carrying a cluster of rRNA genes forming a nucleolar organizer region (NOR). However, we found a substantial interspecific difference in the location of rDNA. While the NOR-bivalent in pachytene nuclei of *L. juvernica* showed the cluster of rRNA genes associated with a large interstitial block of DAPI-positive heterochromatin (inset of Fig. 3a), rDNA occupied a large terminal segment of the NOR-bivalent in *L. reali*. In the latter species, rDNA was not associated with heterochromatin, which was observed at the opposite end of the NOR-bivalent (inset of Fig. 3b).

In *L. sinapis*, we found intraspecific variability in the number and position of rDNA clusters both within and among the offspring of individual females (Fig. 3c-g; hybridization signals of the telomeric probe are not shown). In mitotic metaphase complements from the offspring of one female the 18S rDNA probe localized four rDNA sites at the ends of four middle-sized chromosomes (Fig. 3c), thus indicating two pairs of NOR-chromosomes. However, in mitotic metaphases from the offspring of another female we found either two terminal and one interstitial signal (Fig. 3d) or three terminal hybridization signals (Fig. 3f). The difference among siblings was confirmed in pachytene nuclei where we observed two hybridization signals in a trivalent and one in an element of a bivalent that was heterozygous for rDNA (Fig. 3e) or a pair of signals in a small bivalent and one signal in a larger element of another bivalent (Fig. 3g), respectively.

### Chromosomal location of H3 histone genes

**Fig. 4:**
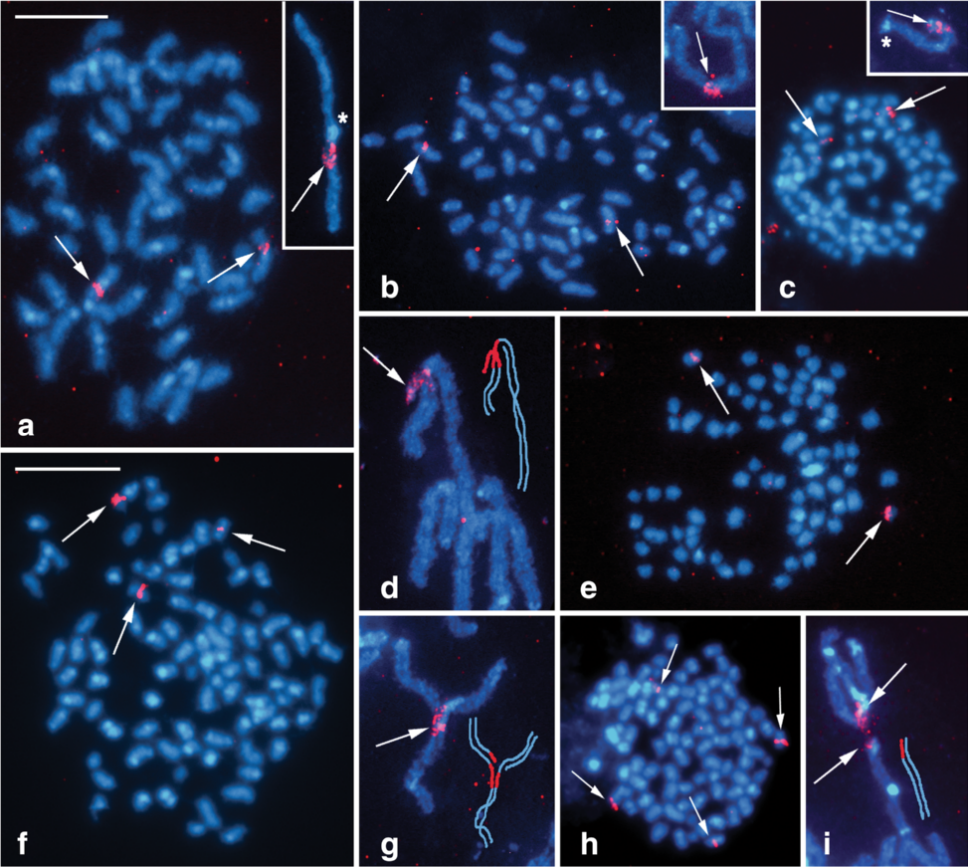
Localization of H3 histone gene clusters in spread chromosome preparations of three *Leptidea* species by FISH with H3 gene probe. Chromosomes were counterstained with DAPI (blue). Arrows indicate hybridization signals of the H3 probe (red); asterisks show DAPI-positive blocks of heterochromatin. **a** Mitotic metaphase of *L. reali* female; the inset in the upper right corner shows the pachytene H3 cluster-carrying bivalent. Figures (**b**–**e**) show intraspecific variability in the location of H3 histone gene clusters in *L. sinapis*: (**b**) mitotic metaphase of male larva; the inset shows the pachytene bivalent carrying a cluster of H3 genes; (**c**) mitotic metaphase of another male from the same offspring; the inset shows the pachytene bivalent carrying a cluster of H3 genes; (**d**) pachytene trivalent observed in the female offspring of another female (schematic drawing shows the structure of the trivalent and positions of two H3 clusters); (**e**) female mitotic metaphase of the same individual. Figures (**f**–**i**) show intraspecific variability in the location of H3 histone gene clusters in *L. juvernica*: (**f**) male mitotic metaphase with three hybridization signals, observed in the vast majority of *L. juvernica* larvae; (**g**) pachytene tetravalent of the same individual (schematic drawing shows the structure of the tetravalent and positions of three H3 clusters); (**h**) mitotic metaphase with four hybridization signals found in one male offspring of another female; (**i**) pachytene tetravalent with three hybridization signals (see schematic drawing in **g**) and bivalent with the fourth hybridization signal located at the end of one homologue. Scale bars = 10 μm; except for (**f**) all images have the same scale

FISH with the H3 histone probe combined with tel-FISH showed constant results only in *L. reali*. In all examined larvae from progenies of three different females we found one interstitial cluster of H3 histone genes in a large pachytene bivalent and two clusters in mitotic metaphase complements (Fig. 4a; hybridization signals of the telomeric probe are not shown). In the pachytene bivalent, the H3 cluster was localized next to a small block of DAPI-positive heterochromatin (inset of Fig. 4a).

In *L. sinapis* and *L. juvernica*, we observed intraspecific variability in the number and location of H3 histone gene clusters both within and among offspring of individual females (Fig. 4b-i; hybridization signals of the telomeric probe are not shown). In some offspring of one *L. sinapis* female we observed an interstitial cluster of H3 histone genes in a long pachytene bivalent (inset of Fig. 4b) corresponding to two hybridization signals in a pair of mitotic chromosomes (Fig. 4b) like in *L. reali*. However, in other offspring of the same female, a single H3 histone gene array mapped to a subterminal region of a short pachytene bivalent (inset of Fig. 4c), corresponding to terminal hybridization signals in a pair of small mitotic chromosomes (Fig. 4c). In the offspring of another *L. sinapis* female, hybridization signals positioned two H3 gene clusters to a pachytene trivalent, one terminal in a short chromosome and the other interstitial in a long chromosome (Fig. 4d). In accordance with this hybridization pattern, the H3 probe identified two mitotic chromosomes, one small and one large (Fig. 4e). In pachytene nuclei of *L. juvernica*, the H3 probe hybridized most often to a tetravalent. We found three clusters of hybridization signals, one terminal in a short element and two interstitial in two long elements of the tetravalent (Fig. 4g). The hybridization pattern was confirmed in mitotic nuclei, where the probe mapped H3 gene arrays to the end of a small chromosome and to the middle of two larger chromosomes (Fig. 4f). The number and location of H3 gene clusters was characteristic for the offspring of four *L. juvernica* females. However, in the offspring of another female we found an additional (fourth) H3 gene cluster located at the end of one element of a pachytene bivalent (Fig. 4i), corresponding to a total number of four hybridization signals in mitotic nuclei (Fig. 4h).

### Sex chromosome constitution

We first examined the presence or absence of female specific sex chromatin in polyploid somatic nuclei of all three *Leptidea* species. The sex chromatin consists of multiple copies of the W chromosome, which usually form one conspicuous heterochromatin body in somatic interphase nuclei of lepidopteran females [44]. In the majority of female larvae of all three species, we observed one larger, more intensely stained heterochromatin body and two tiny indistinct bodies (Additional file 2: Figure S2a). Yet the larger body was much smaller in comparison to the sex chromatin typically observed in females of other lepidopteran species (*cf*. [45, 46]). In other females we found a variable number of tiny heterochromatin bodies ranging from none to four. Similar findings were made in branched nuclei of adult females with a higher level of ploidy (Additional file 2: Figure S2b). In the majority of *Leptidea* males, no sex chromatin was observed in polyploid cells (Additional file 2: Figure S2c). However, in a few male specimens we found a tiny heterochromatin body of uncertain origin (Additional file 2: Figure S2d). The small size and fragmentation of sex chromatin in *Leptidea* females indicate the presence of interchromosomal rearrangements involving the W chromosome (see [42, 47]).

**Fig. 5:**
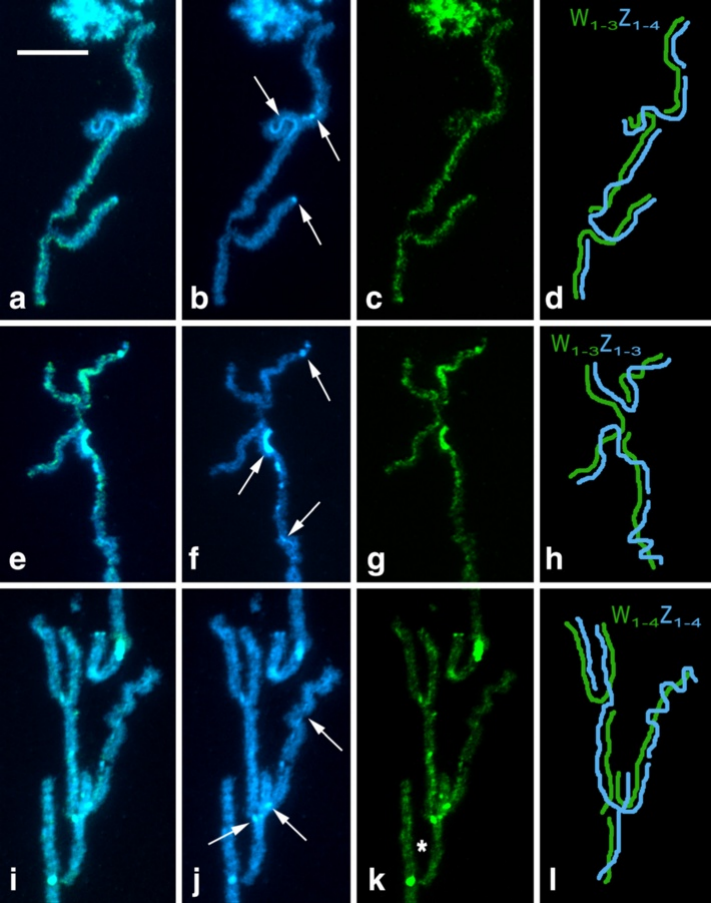
Genomic *in situ* hybridization (GISH) in pachytene oocytes of *Leptidea juvernica* (**a–d**), *L. sinapis* (**e–h**) and *L. reali* (**i–l**). Female-derived genomic probes were labelled with fluorescein-12-dUTP (green) and chromosomes were counterstained with DAPI (blue). Figures (**a**–**d**), (**e**–**h**) and (**i**–**l**) show detailed analyses of sex chromosome multivalents W_1-n_Z_1-n_: (**a**, **e**, **i**) merged images of female genomic probes and DAPI staining; (**b**, **f**, **j**) DAPI images; arrows indicate DAPI-positive W-chromosome segments and heterochromatic blocks at the end of the W chromosomes; (**c**, **g**, **k**) hybridization pattern of the female genomic probes; the asterisk indicates an undifferentiated segment of one of the W chromosomes; (**d**, **h**, **l**) schematic drawings of the sex chromosome multivalents. Scale bar = 10 μm

To identify the W chromosome we examined spread preparations of pachytene oocytes using a combination of GISH and tel-FISH. While GISH differentiated the W chromosome thread in female pachytene nuclei, the telomeric probe helped us to determine chromosomal ends. The female-derived genomic probe also hybridized to heterochromatin blocks on autosomes, which made the identification of the W chromosome more difficult in pachytene nuclei and impossible in mitotic metaphases. Nevertheless, the analysis revealed multiple sex chromosomes in all three *Leptidea* species with 3–4 W and 3–4 Z chromosomes (Fig. 5a–l; hybridization signals of the telomeric probe are shown in Additional file 3: Figure S3a–l). In *L. juvernica*, we observed a W_1_W_2_W_3_Z_1_Z_2_Z_3_Z_4_ sex chromosome constitution (Fig. 5a–d). While the female genomic DNA (gDNA) probe strongly bound to two W chromosomes, the third one was highlighted only partially (Fig. 5c). Moreover, two of the three W chromosomes were partially differentiated by DAPI-positive heterochromatin (Fig. 5b, two upper arrows). We found only a small heterochromatin block at the very end of the third W chromosome (Fig. 5b, the lower arrow). In *L. sinapis*, we found a W_1_W_2_W_3_Z_1_Z_2_Z_3_ sex chromosome system (Fig. 5e–h) with an intensely stained block of heterochromatin on one of the W chromosomes (Fig. 5f, the middle arrow). We also found a small heterochromatin block at the very end of the smallest W chromosome (Fig. 5f, the upper arrow) while the third W chromosome was discernible only due to hybridization signals of the female gDNA probe (Fig. 5g). In the third species, *L. reali*, the sex chromosome constitution was W_1_W_2_W_3_W_4_Z_1_Z_2_Z_3_Z_4_ (Fig. 5i–l). Except for the smallest W, the W chromosomes were highlighted with the female gDNA probe (Fig. 5k), but the staining pattern of DAPI was indistinctive with only few small heterochromatin blocks of higher intensity (Fig. 5j, arrows).

**Fig. 6:**
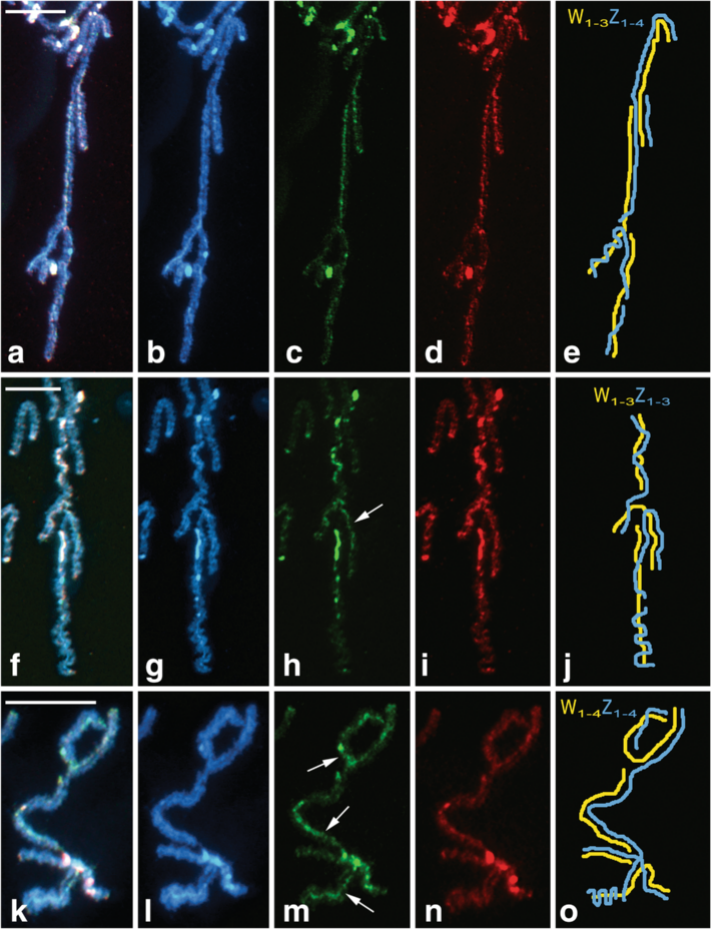
Comparative genomic hybridization (CGH) in pachytene oocytes of *Leptidea juvernica* (**a–e**), *L. sinapis* (**f–j**) and *L. reali* (**k–o**). Female-derived genomic probes were labelled with fluorescein-12-dUTP (green), male-derived genomic probes were labelled with Cy3-dUTP (red) and chromosomes were counterstained with DAPI (blue). Figures (**a**–**e**), (**f**–**j**) and (**k**–**o**) show detailed analyses of sex chromosome multivalents W_1-n_Z_1-n_: (**a**, **f**, **k**) merged images of both genomic probes and DAPI staining; (**b**, **g**, **l**) DAPI images; (**c**, **h**, **m**) female genomic probes; arrows indicate W-chromosome segments with female-specific hybridization pattern; (**d**, **i**, **n**) male genomic probes; (**e**, **j**, **o**) schematic drawings of the sex chromosome multivalents. Scale bars = 10 μm

The level of molecular differentiation of the W and Z chromosomes was examined using CGH. In pachytene oocytes of the three *Leptidea* species, the WZ multivalent was discernible from autosomes due to stronger binding of both female- and male-derived probes to the W chromosomes (Fig. 6a–o). A detailed analysis of the WZ multivalent at the pachytene stage of *L. juvernica* revealed a similar labelling pattern of both probes (Fig. 6a–e). In *L. sinapis*, the W chromosomes were decorated with strong but scattered hybridization signals of both genomic probes (Fig. 6f, h, i) with a slight preference for the female probe (Fig. 6h). The highest level of molecular differentiation of the W and Z chromosomes was observed in *L. reali* (Fig. 6k–o), where three out of four W chromosomes were preferentially labelled by the female-derived probe (Fig. 6m). However, the smallest W chromosome was almost indistinguishable from the Z chromosome (Fig. 6m–o). Hybridization signals of the male-derived genomic probe were considerably weaker, except for a few intense heterochromatin blocks located on one W chromosome (Fig. 6n).

## Discussion

We performed a detailed karyotype analysis of three cryptic *Leptidea* species (*L. juvernica*, *L. sinapis* and *L. reali*) by means of standard and molecular cytogenetic techniques. Previous studies showed both inter- and intraspecific variation in chromosome numbers in all three studied species. However, the results were based on chromosome counts from squash preparations of metaphase I spermatocytes [38, 39], which did not allow the analysis of complex meiotic figures such as multivalents. Using FISH with (TTAGG)_*n*_ telomeric probes, we confirmed the presence of numerous multivalents in female pachytene nuclei as well as in male pachytene and metaphase I complements in all three species. Detailed analysis of male and female mitotic metaphases prepared from wing imaginal discs allowed us to determine more accurately the range of diploid chromosome numbers that were 2n = 51–55 in *L. reali* (Spanish population), 2n = 69–73 in *L. sinapis* and 2n = 85–91 in *L. juvernica* (both Czech populations). These numbers are broadly in line with previous findings [38, 39]. Nevertheless, we further extended the range of chromosome numbers observed in *L. juvernica* and *L. reali* and provided data from new localities for *L. juvernica* and *L. sinapis*.

Besides inter- and intraspecific karyotype variability, the analysis of mitotic chromosomes allowed us to identify differences in chromosome numbers even within offspring of individual females of all three species. Although such intra-population variability could be caused by the presence of supernumerary chromosomes, *i.e.* B-chromosomes, this scenario was deemed unlikely in the case of *Leptidea* species [39]. Yet, we cannot totally exclude the involvement of B-chromosomes, especially as they were observed in related white butterflies from the family Pieridae [34]. In wild silkworms (*Samia cynthia*), chromosomal polymorphism among geographical populations/subspecies was ascribed to repeated autosome-sex chromosome fusions resulting in neo-sex chromosomes and reduced chromosome numbers [21]. Similar intraspecific variation resulting in reduced chromosome numbers was also reported in grasshoppers (*e.g.* [48]) and mammals (*e.g.* [49, 50]), mainly as a result of Robertsonian translocations. However, karyotype variation observed in the three *Leptidea* species surpasses previous reports. Our findings, namely the relatively low number of large chromosomes in *L. reali*, variability in chromosome size in the other two species and the occurrence of multivalents in meiotic nuclei of all three species, suggest that the *Leptidea* karyotypes are differentiated by multiple chromosome fusions and fissions. In addition, our preliminary data showing a similar size of interphase nuclei in the *Leptidea* species studied (Additional file 4: Figure S4) suggest that their karyotypes did not differentiate through polyploidy. Uneven chromosome segregation of multivalents during meiotic division is thus the most plausible explanation for the intraspecific and intra-population karyotype variations.

The karyotypes of *Leptidea* species also differed in the number and location of two cytogenetic markers used in this study, clusters of rRNA genes (major rDNA) and H3 histone genes. In *L. reali*, the species with the lowest chromosome number, all larvae showed consistent results with a single terminal rDNA cluster and an interstitial cluster of H3 genes per haploid genome. The highest variability in the number and position of both cytogenetic markers was observed within and among the offspring of individual *L. sinapis* females. In *L. juvernica*, the number and location of H3 histone genes differed in the progeny of individual females, while one rDNA cluster was always located in the middle of one pachytene bivalent. Except for *L. reali*, both cytogenetic markers often marked multivalents. Interspecific differences as well as intrapopulation variability in rDNA distribution in *L. sinapis* are in agreement with the hypothesis on dynamic evolution of genes for major RNAs in Lepidoptera [51]. However, the differences in the number and location of H3 histone genes in *L. sinapis* and especially *L. juvernica* are rather surprising, since this marker shows a highly conserved pattern in the lepidopteran family Tortricidae [52] and other insect groups, such as the Acrididae grasshoppers [53] and Scarabaeinae beetles [54]. Our results thus support the previously reported intraspecific variability in the karyotype of *L. sinapis* [39] and highlight the ongoing explosive karyotype evolution in all three *Leptidea* species.

To further explore *Leptidea* karyotypes and to identify their sex chromosomes we performed GISH combined with tel-FISH. In pachytene nuclei of lepidopteran females, the WZ bivalent is usually easily discernible with GISH by deep staining of the W chromosome thread with the fluorescently labelled female gDNA probe [43, 45, 55]. In all three *Leptidea* species, the analysis revealed unique sex chromosome systems with the following constitutions: W_1_W_2_W_3_Z_1_Z_2_Z_3_Z_4_ in *L. juvernica*, W_1_W_2_W_3_Z_1_Z_2_Z_3_ in *L. sinapis* and W_1_W_2_W_3_W_4_Z_1_Z_2_Z_3_Z_4_ in *L. reali*. The constitution seemed to be stable in the progenies of individual females. Multiple sex chromosome systems have been documented in mammals [56, 57], fish [58, 59] and spiders [60, 61]. However, the majority of moths and butterflies show a WZ/ZZ sex chromosome system. Multiple sex chromosomes have so far been found only in seven genera and only in two different constitutions, either with W_1_W_2_Z or WZ_1_Z_2_ trivalents in females [46]. Thus, the sex chromosome constitutions observed in *Leptidea* stand out for its complexity and the number of chromosomes involved in the multivalent in meiosis. In addition, this is the first case of multiple sex chromosomes in butterflies (Papilionoidea). Besides sex chromosomes, the gDNA probes also highlighted heterochromatin blocks abundantly present in the karyotypes of all three studied species. In Lepidoptera, heterochromatin is usually confined to the NOR containing rDNA repeats [51, 52] and to the W chromosome [44, 62]. So far, similar heterochromatin blocks have been found only in subtelomeric regions of the white butterfly *Pieris brassicae* (Pieridae) [51] and on chromosome 24 of *Bombyx mori* [63]. In *Leptidea*, however, heterochromatin was evenly distributed throughout the whole genome. Such distribution suggests the preponderance of tandemly arranged repetitive sequences in *Leptidea* genomes in comparison with other lepidopteran species, which could ultimately contribute to the remarkable karyotype diversity in this group.

In addition to the different sex chromosome constitution, the three *Leptidea* species also differed in their overall genomic hybridization pattern. In *L. sinapis* and *L. juvernica*, all W chromosomes were at least partially differentiated by strong binding of fluorescently labelled gDNA probes with GISH and CGH, indicating the accumulation of repetitive sequences and transposable elements in the W chromosomes (*cf*. [64]). In *L. reali*, one of the W chromosomes was not highlighted by any gDNA probe. This W chromosome probably represents an evolutionarily young element, which did not have sufficient time to differentiate. Individual Z chromosomes involved in multivalents thus probably correspond to the so-called evolutionary strata, which were also reported in mammals, birds and plants [65–69]. Moreover, similar hybridization patterns of male and female genomic probes in CGH experiments suggest a predominance of common repetitive sequences and transposons and a low amount of W-specific sequences on the W chromosomes of *L. sinapis* and *L. juvernica*. On the contrary, the preferential binding of the female-derived genomic probe to three of the four W chromosomes in *L. reali* suggest a relatively high proportion of W-specific sequences.

Observed differences in chromosome numbers and location of the major rDNA and H3 histone gene clusters as well as the existence of complex sex chromosome systems corroborate the role of chromosomal rearrangements in the speciation of the closely related *Leptidea* species examined in this study. It has been shown that chromosomal rearrangements have a potential to limit gene flow and thus facilitate the development and maintenance of reproductive isolation by means of suppressed recombination [16–18]. The majority of studies on the effects of chromosome fusion and fission on speciation have been done in organisms with monocentric chromosomes that exhibit Robertsonian translocations, *i.e.* centric fusions [70–72]. These studies confirmed the role of chromosomal fusions in reducing the frequency of recombination. The variation in chromosome size and number is explained as a result of frequent fusion and fission events also in taxa with holokinetic chromosomes [39, 73], in which kinetochores are distributed along most of the poleward facing chromosome surface [74]. In this case, fusion is likely to behave as a stable centric fusion and fission leads to viable chromosomal fragments that are normally inherited during meiosis [75, 76]. A recent study stressed the effect of chromosome fusion on the recombination rate in holokinetics [77]. Moreover, studies in sedges (*Carex*, Cyperaceae) proved that fusion and fission of holokinetic chromosomes also have the potential to restrict gene flow and lead to divergence and eventually speciation [78].

The complex sex chromosome constitution revealed in this study is likely another factor involved in the speciation of *Leptidea* butterflies. It has been proposed that the Z sex chromosome could play a disproportionately larger role in adaptive evolution compared to autosomes [79–81]. This so-called ‘large-Z effect’ was reported in both birds [79, 81–83] and Lepidoptera ([5, 84], the two largest taxa with female heterogamety. Furthermore, detailed studies on the neo-sex chromosome evolution in geographic populations of *S. cynthia* and leaf-rollers of the family Tortricidae suggest that sex chromosomal rearrangements play a major role in the formation of reproductive barriers between populations and contribute to radiation in some lepidopteran taxa, respectively [21, 85]. In *Leptidea*, the multiple sex chromosome system most likely originated by complex translocations between the ancestral WZ pair and several autosomes, which increased the number of sex-linked genes and thus accelerated the accumulation of genetic incompatibilities among populations. This is supported by the intraspecific stability of their multiple sex chromosomes systems, which is in stark contrast to the evolutionary dynamics of their autosomes. Another signal of reinforcement could be the fact that the most recently diverged sister species, *L. sinapis* and *L. reali*, display not only the largest differences in chromosome numbers in sympatry [38, 39] but also the most different sex determination system (as shown in this study).

## Conclusions

To conclude, we confirmed significant differences in the number and structure of chromosomes within and among closely related wood white butterflies. We showed that the distribution of cytogenetic markers differs remarkably even in the offspring of individual females, probably due to irregular segregation of multivalents in meiosis. Our results suggest rapid karyotype evolution in the examined *Leptidea* species and stress the role of chromosomal rearrangements, especially multiple chromosome fusions and fissions, in their speciation. Remarkably, all three *Leptidea* species have complex sex chromosome systems with 3–4 W and 3–4 Z chromosomes. Such sex chromosome constitutions are unique among Lepidoptera and should be counted as an additional factor potentially contributing to the speciation process in *Leptidea* butterflies. Taken together, these findings add to accumulating evidence on the important role of chromosomal rearrangements in speciation and also point to the relevance of multiple sex chromosomes in species divergence and the formation of reproductive barriers.

## Methods

### Sample collecting

Fresh adult specimens of *Leptidea juvernica* and *L. sinapis* were collected in the Czech Republic, namely in the surroundings of České Budějovice and near Havraníky village in the Podyjí National Park in South Moravia, respectively. The third species, *L. reali*, was collected in the Montseny area near Barcelona, Spain. In the laboratory, fertilized females were kept in plastic containers to lay eggs. The bodies of all collected individuals were then placed into 1.5 ml Eppendorf tubes, frozen in liquid nitrogen and stored at −80 °C until DNA extraction, except for their genitalia which were immediately used for morphometric analysis. Hatched larvae were reared on corresponding host plants, *Lathyrus pratensis* for *L. juvernica* and *L. reali* and *Securigera varia* for *L. sinapis*, at room temperature and normal day/night regime.

### Genitalia preparation and morphometric analysis

Male and female genitalia were dissected in a physiological solution and inspected under a stereomicroscope. Lengths of two elements of the male genitalia, *phallus* and *saccus* and one element of the female genitalia, *ductus bursae*, were measured. These diagnostic characters discriminate *L. sinapis* from the other two species, *L. juvernica* and *L. reali*, which cannot be reliably distinguished from each other based on morphological features (Fig. 1; [38]).

### Specimen sequencing

Genomic DNA (gDNA) was extracted from legs of every female that gave progeny used in cytogenetic studies, *i.e.* from 6 *L. sinapis*, 6 *L. reali* and 4 *L. juvernica* females, using the NucleoSpin Tissue XS kit (Macherey-Nagel, Düren, Germany) according to the supplier’s protocol. To confirm the taxonomic determination of the examined specimens, molecular phylogenetic trees were constructed using one mitochondrial gene, cytochrome *c* oxidase subunit 1 (*COI*) and one nuclear marker, the internal transcribed spacer 2 (*ITS2*). For each individual, a partial sequence of both markers was amplified by polymerase chain reaction (PCR) using two pairs of primers: for *COI* (658 bp) LepF1 (5′-ATTCAACCAATCATAAAGATATTGG-3′) and LepR1 (5′-TAAACTTCTGGATGTCCAAAAAATCA-3′); for *ITS2* (684 bp) ITS3 (5′-GCATCGATGAAGAACGCAGC-3′) and ITS4 (5′-TCCTCCGCTTATTGATATGC-3′) [38].

PCR was carried out in 25-μL reaction volumes containing 1× Ex *Taq* buffer (TaKaRa, Otsu, Japan), 0.2 mM dNTP mix, 5 μmol of each primer, 0.25 U Ex *Taq* Hot Start DNA polymerase (TaKaRa) and about 100 ng of template gDNA. The typical thermal cycling profile for *COI* consisted of an initial denaturation period of 5 min at 95 °C followed by 30 cycles of 30 s at 95 °C, 1 min at 44 °C and 1 min at 72 °C and by a final extension step of 7 min at 72 °C. The profile was similar for the nuclear marker *ITS2* except for the annealing temperature, which was 50 °C. PCR products were purified using a Wizard SV Gel and PCR Clean-Up System (Promega, Madison, WI, USA) and sequenced using BigDye® Terminator v3.1 Cycle Sequencing Kit (Applied Biosystems, Foster City, CA, USA).

### Phylogenetic analysis

Sequences were edited and aligned using GENEIOUS PRO 4.7.5 created by Biomatters (http://www.geneious.com/). Our sequences were combined with all available *COI* and *ITS2* haplotypes of *Leptidea sinapis, L. reali* and *L. juvernica* identified in a previous study [41] and with sequences of *L. morsei*, *L. amurensis*, *L. lactea* and *L. duponcheli* that were used as outgroup (Additional file 5: Table S1). Thus, the final *COI* alignment contained 69 nucleotide sequences and was 658 bp long, while the *ITS2* alignment involved 28 sequences and consisted of 684 positions.

To confirm the identification of the examined specimens, neighbor-joining trees [86] were built for *COI* and *ITS2*. Both trees were based on *p*-distance [87] and pairwise deletion. Node supports were assessed through 100 bootstrap replicates [88]. The trees were inferred in MEGA6 [89].

### Chromosome preparation

In each *Leptidea* species, two types of spread chromosome preparations were made from fifth instar male and female larvae. Mitotic chromosomes were obtained from wing imaginal discs characterized by a high mitotic index [52], while meiotic chromosomes were obtained from ovaries and testes. In both cases we used the procedure described in [45]. All preparations were passed through a graded ethanol series (70 %, 80 % and 100 %, 1 min each) and stored at −80 °C until further use.

### Preparation of polyploid nuclei

Malpighian tubules were dissected out from fifth instar larvae of both sexes and adult females in a physiological solution. Removed tubules were fixed in ethanol/chloroform/acetic acid (6:3:1) for 1 minute and stained in 1.5 % lactic acetic orcein. Preparations were inspected under a light microscope for the presence of female specific sex chromatin [44].

### FISH with fluorochrome-labelled probes

For the chromosome counts we used spread chromosome preparations from wing imaginal discs stained by FISH with (TTAGG)_*n*_ telomeric probes (tel-FISH), which helped us to identify the chromosome ends. The telomeric probes were generated by non-template PCR as described in [90] and labelled by Cy3-dUTP (GE Healthcare, Milwaukee, WI, USA) using a Nick Translation Kit (Abbott Molecular Inc., Des Plaines, IL, USA) with 1 hour incubation at 15 °C. For tel-FISH we followed the procedure described in [55]. The probe cocktail contained 100 ng of Cy3-labelled telomeric probe and 25 μg of sonicated salmon sperm DNA (Sigma-Aldrich, St. Louis, MO, USA) in 10 μl of 50 % formamide and 10 % dextran sulfate in 2× SSC.

GISH and CGH were used to identify the sex chromosomes and examine their molecular differentiation [43, 91]. GISH was combined with tel-FISH for better resolution of the sex chromosome constitution [55]. Genomic DNAs for both GISH and CGH experiments were extracted separately from adult *Leptidea* males and females by standard phenol-chloroform procedure. Male gDNA was also amplified by GenomiPhi HY DNA Amplification Kit (GE Healthcare), thereafter sonicated using a Sonopuls HD 2070 (Bandelin Electric, Berlin, Germany) and used as a competitor DNA [52]. The extracted male gDNA was labelled with Cy3-dUTP (GE Healthcare) and female gDNA with fluorescein-12-dUTP (Invitrogen, Carlsbad, CA, USA) using the Nick Translation Kit with 8 hours incubation at 15 °C.

For GISH combined with tel-FISH the probe cocktail contained fluorescein-labelled female gDNA (300 ng), Cy3-labelled telomeric probe (100 ng), unlabelled sonicated male gDNA (3 μg) and sonicated salmon sperm DNA (25 μg). The probe cocktail for CGH was similar to GISH, except that it contained Cy3-labelled male gDNA (300 ng) instead of the telomeric probe. The preparations were counterstained with 0.5 mg/mL DAPI and mounted in antifade based on DABCO (Sigma-Aldrich).

### FISH with biotin- and digoxigenin-labelled probes

Unlabelled 18S rDNA probe was generated by PCR from the codling moth (*Cydia pomonella*) gDNA extracted from adults by standard phenol-chloroform procedure as described in [43]*.* The probe was labelled with biotin-16-dUTP (Roche Diagnostics GmbH, Mannheim, Germany) by nick translation using the Nick Translation Kit with 1 hour and 45 minutes incubation at 15 °C.

Unlabelled H3 histone probe was obtained by PCR from *L. sinapis* gDNA. PCR was carried out using degenerate forward (5′-ATGGCNCGTACNAARCARAC-3′) and reverse (5′-TANGCACGYTCNCGGAT-3′) primers and the final PCR product was cloned as described in [52]. The probe was labelled in 25-μL PCR reaction containing 1× Ex *Taq* buffer, 0.1 mM dATP, dGTP and dCTP, 0.065 mM dTTP, 0.035 mM biotin-16-dUTP, 5 μmol of each M-13 universal primers, 0.25 U TaKaRa Ex *Taq* Hot Start DNA polymerase and about 5 ng of plasmid DNA. The thermal cycle profile consisted of an initial denaturation period of 2 min at 94 °C followed by 30 cycles of 30 s at 94 °C, 30 s at 57 °C and 1 min at 72 °C and a final extension step of 2 min at 72 °C.

In FISH experiments, 18S rDNA and H3 histone probes were combined with telomeric probes. Unlabelled telomeric probe generated by non-template PCR (see above) was labelled with digoxigenin (Roche Diagnostics GmbH) using the Nick Translation Kit. The detection of biotin was carried out as described in [43]: the signals were detected with Cy3-conjugated streptavidin (Jackson ImmunoRes. Labs. Inc., West Grove, PA, USA), amplified with biotinylated anti-streptavidin (Vector Labs. Inc., Burlingame, CA, USA) and again detected with Cy3-conjugated streptavidin. The detection of digoxigenin was carried out by Fluorescent Antibody Enhancer Set for DIG Detection (Roche Diagnostics GmbH). Like in the above-mentioned FISH experiments, the preparations were counterstained with 0.5 μg/mL DAPI and mounted in the DABCO-based antifade.

### Microscopy and image processing

Preparations from FISH experiments were observed under a Zeiss Axioplan 2 microscope (Carl Zeiss Jena, Germany). Black-and-white images were recorded with a cooled F-View CCD camera using AnalySIS software, version 3.2 (Soft Imaging System GmbH, Münster, Germany). In all preparations, images were captured separately for each fluorescent dye, pseudocoloured (light blue for DAPI, green for fluorescein and red for Cy3) and superimposed with Adobe Photoshop, version 7.0.

## Additional files

Additional file 1: Figure S1. Neighbor-joining tree of nuclear *ITS2* haplotypes of *L. sinapis* (grey background), *L. reali* (orange background) and *L. juvernica* (blue background). Specimens sequenced and analysed in this study are indicated by an asterisk. *Leptidea amurensis*, *L. lactea*, *L. morsei* and *L. duponcheli* were used as outgroup. For the origin of all specimens and GenBank accession numbers, see Additional file 5: Table S1. The scale represents 0.01 substitutions per site. Bootstrap supports (100 replicates) are shown next to the recovered nodes. Additional file 2: Figure S2. The status of sex chromatin in polyploid nuclei of three *Leptidea* species. The orcein-stained preparations were made from Malpighian tubule cells of the fifth instar larvae (**a**, **c**, **d**) and adult females (**b**). Black arrows indicate a larger deeply stained heterochromatin body, while arrowheads show smaller bodies. (**a**) A lower-ploidy female nucleus of *L. sinapis* with one larger and two smaller bodies. (**b**) A highly polyploid female nucleus of *L. sinapis* with two bodies, one larger and one smaller. (**c**) A male nucleus of *L. reali* without distinguishable heterochromatin bodies. (**d**) A male nucleus of *L. reali* with one smaller body. Scale bar = 10 μm. Additional file 3: Figure S3. Analysis of sex chromosome multivalents of pachytene oocytes in *Leptidea juvernica* (a–d), *L. sinapis* (e–h) and *L. reali* (i–l) using FISH with the (TTAGG)_*n*_ telomeric probe. Hybridization signals of the Cy3-dUTP-labelled telomeric probe (red) indicate chromosome ends. Chromosomes were counterstained with DAPI (blue). Figures (**a**–**d**), (**e**–**h**) and (**i**–**l**) show sex chromosome multivalents W_1-n_Z_1-n_: (**a**, **e**, **i**) merged images of the (TTAGG)_*n*_ telomeric probe and DAPI staining; (**b**, **f**, **j**) DAPI images; note DAPI-highlighted heterochromatic segments of the W chromosomes; (**c**, **g**, **k**) hybridization pattern of the (TTAGG)_*n*_ telomeric probe; (**d**, **h**, **l**) schematic drawings of the sex chromosome multivalents; yellow dots indicate the ends of individual chromosomes involved in the multivalents. Scale bar = 10 μm. Additional file 4: Figure S4. Comparison of interphase nuclei sizes in three *Leptidea* species. The y-axis shows the number of pixels. Micrographs of interphase nuclei were taken from DAPI-stained spread preparations of wing discs from three different larvae of each *Leptidea* species, using the same resolution. In these micrographs, we measured the area of 144 nuclei of *L. juvernica*, 154 nuclei of *L. reali* and 130 nuclei of *L. sinapis*. The measurements were carried out using the software JMicroVision v1.2.7 [Roduit N: JMicroVision: Image analysis toolbox for measuring and quantifying components of high-definition images. Version 1.2.7. http://www.jmicrovision.com (accessed 27 March 2015)]. Calibration was performed using an image resolution so that the area of each nucleus was measured in pixels. The average size of nuclei was calculated for each species independently and then compared between species by one-way ANOVA using the software Statistica for Windows, version 8.0 (StatSoft, Inc., Tulsa, OK, USA). The comparison of interphase nuclei revealed no statistically significant between-species differences in their size (*F*_(2, 9)_ = 0.6782; *P* = 0.5425). The mean (± S.E.) area of interphase nuclei was 22434 ± 2296 pixels for *L. juvernica*, 19781 ± 1965 pixels for *L. reali* and 19835 ± 1021 pixels for *L. sinapis*. Additional file 5: Table S1. List of specimens included in phylogenetic analyses. Sequences obtained in this study are in blue, the other sequences were downloaded from GenBank and are representative for all the *COI* and *ITS2* haplotypes of *Leptidea sinapis*, *L. reali* and *L. juvernica* identified in a previous study [41]. The haplotype numbers correspond to those in [41].

## Abbreviations

CGH: Comparative genomic hybridization
*COI*: Cytochrome *c* oxidase subunit 1
FISH: Fluorescence *in situ* hybridization
gDNA: Genomic DNA
GISH: Genomic *in situ* hybridization
H3: Histone H3
*ITS2*: Internal transcribed spacer 2
MI: Meiotic metaphase I
NOR: Nucleolar organizer region
PCR: Polymerase chain reaction
rDNA: Ribosomal DNA
rRNA: Ribosomal RNA
tel-FISH: Fluorescence *in situ* hybridization with (TTAGG)_*n*_ telomeric probe
