# Supplementary material for: Dynamic karyotype evolution and unique sex determination systems in Leptidea wood white butterflies
Source: BMC Evol Biol. 2015 May 19;15:89. doi: 10.1186/s12862-015-0375-4 (PMC4436027; doi:10.1186/s12862-015-0375-4)
Supplement: Additional file 1: Figure S1. — Neighbor-joining tree of nuclear ITS2 haplotypes of L. sinapis (grey background), L. reali (orange background) and L. juvernica (blue background). Specimens sequenced and analysed in this study are indicated by an asterisk. Leptidea amurensis, L. lactea, L. morsei and L. duponcheli were used as outgroup. For the origin of all specimens and GenBank accession numbers, see Additional file 5: Table S1. The scale represents 0.01 substitutions per site. Bootstrap supports (100 replicates) are shown next to the recovered nodes. [file 12862_2015_375_MOESM1_ESM.pdf]

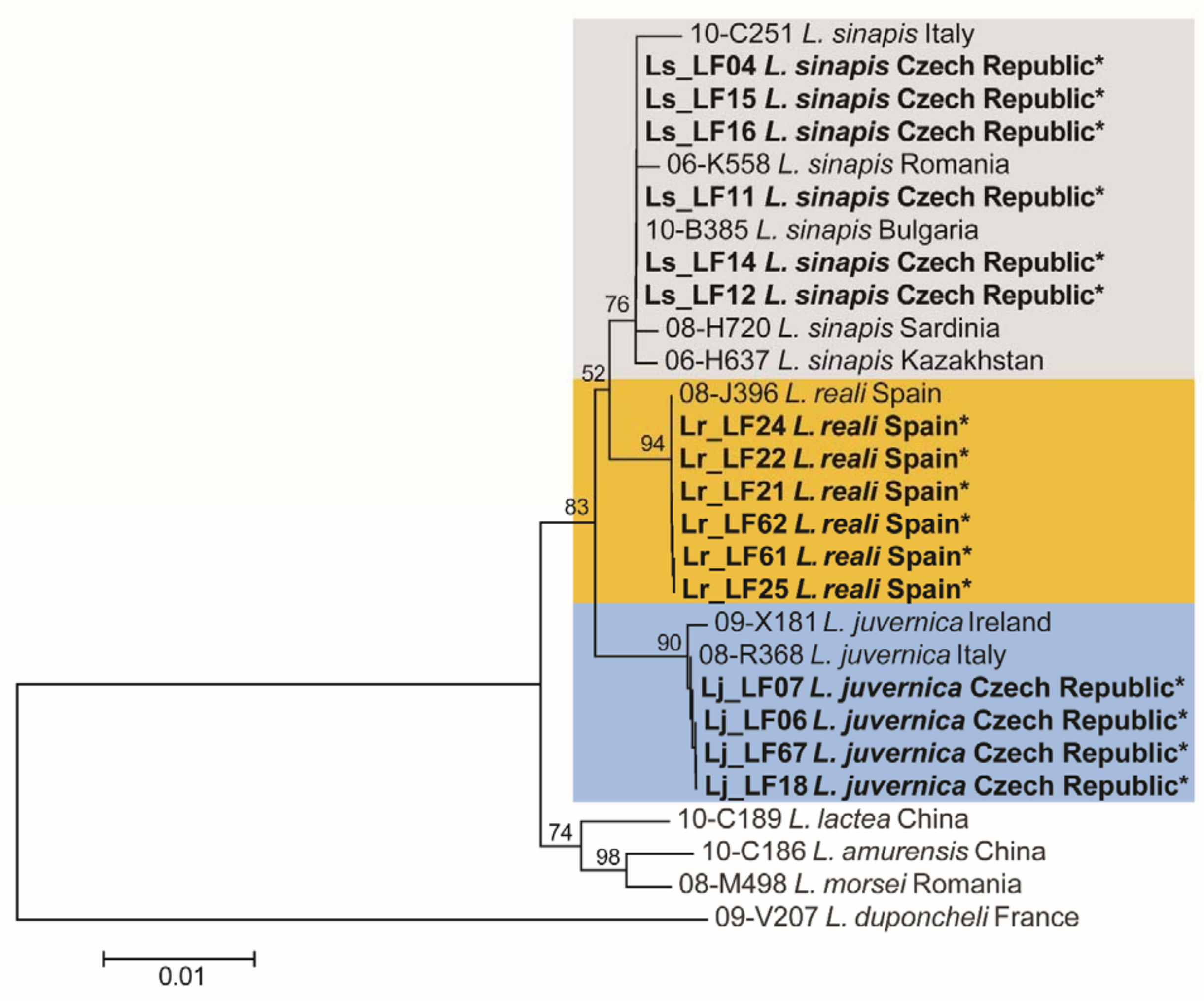

Figure S1 Neighbor-joining tree of nuclear *ITS2* haplotypes of *L. sinapis* (grey background), *L. reali* (orange background), and *L. juvernica* (blue background). Specimens sequenced and analysed in this study are indicated by an asterisk. *Leptidea amurensis*, *L. lactea*, *L. morsei*, and *L. duponcheli* were used as outgroup. For the origin of all specimens and GenBank accession numbers, see Additional file 5: Table S1. The scale represents 0.01 substitutions per site. Bootstrap supports (100 replicates) are shown next to the recovered nodes.
