# Supplementary material for: Dynamic karyotype evolution and unique sex determination systems in Leptidea wood white butterflies
Source: BMC Evol Biol. 2015 May 19;15:89. doi: 10.1186/s12862-015-0375-4 (PMC4436027; doi:10.1186/s12862-015-0375-4)
Supplement: Additional file 2: Figure S2. — The status of sex chromatin in polyploid nuclei of three Leptidea species. The orcein-stained preparations were made from Malpighian tubule cells of the fifth instar larvae (a, c, d) and adult females (b). Black arrows indicate a larger deeply stained heterochromatin body, while arrowheads show smaller bodies. (a) A lower-ploidy female nucleus of L. sinapis with one larger and two smaller bodies. (b) A highly polyploid female nucleus of L. sinapis with two bodies, one larger and one smaller. (c) A male nucleus of L. reali without distinguishable heterochromatin bodies. (d) A male nucleus of L. reali with one smaller body. Scale bar = 10 μm. [file 12862_2015_375_MOESM2_ESM.pdf]

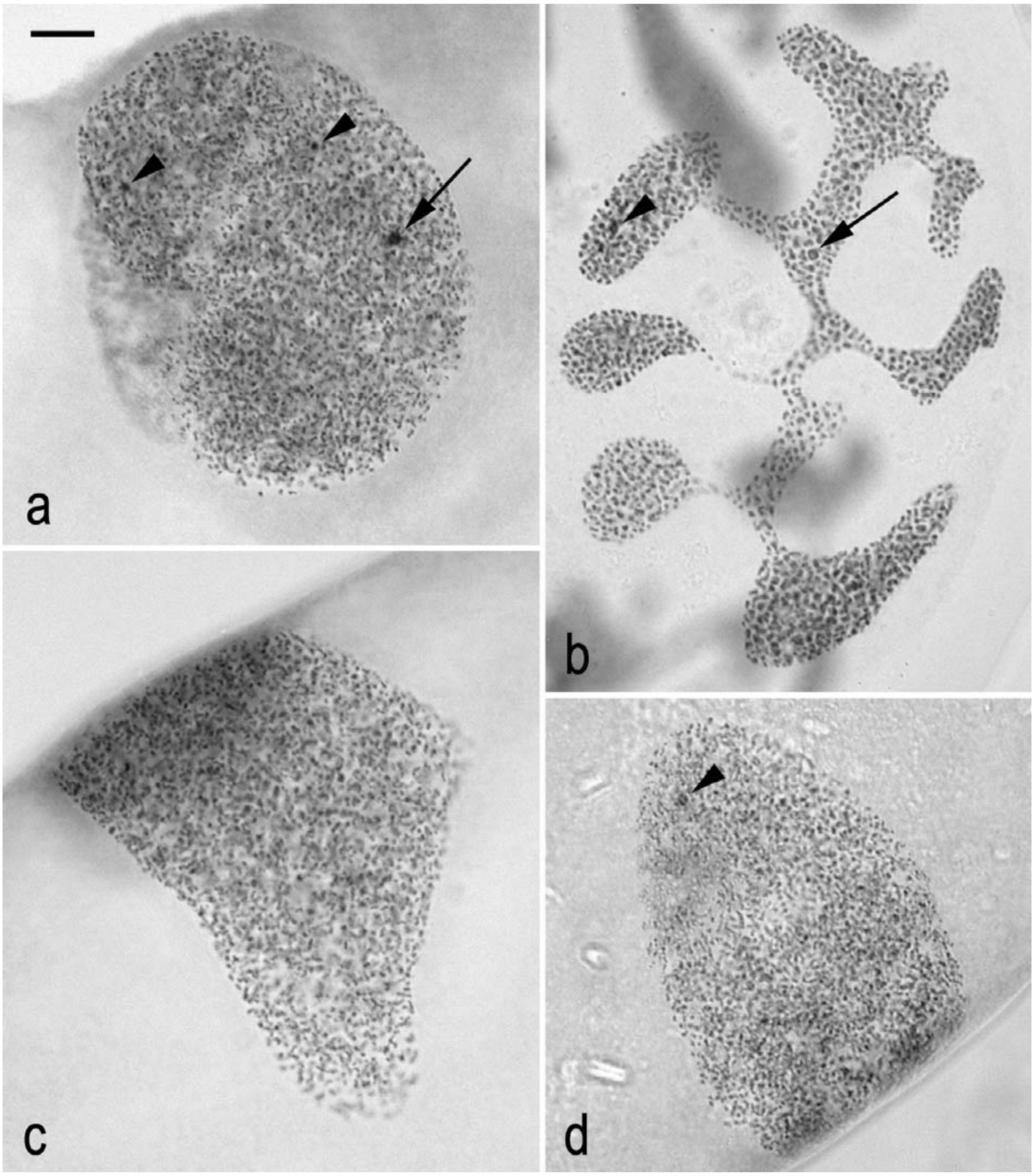

Figure S2 The status of sex chromatin in polyploid nuclei of three *Leptidea* species. The orcein-stained preparations were made from Malpighian tubule cells of the fifth instar larvae (**a**, **c**, **d**) and adult females (**b**). Black arrows indicate a larger deeply stained heterochromatin body, while arrowheads show smaller bodies. (**a**) A lower-ploidy female nucleus of *L. sinapis* with one larger and two smaller bodies. (**b**) A highly polyploid female nucleus of *L. sinapis* with two bodies, one larger and one smaller. (**c**) A male nucleus of *L. reali* without distinguishable heterochromatin bodies. (**d**) A male nucleus of *L. reali* with one smaller body. Scale bar = 10 µm.
