# Supplementary material for: Dynamic karyotype evolution and unique sex determination systems in Leptidea wood white butterflies
Source: BMC Evol Biol. 2015 May 19;15:89. doi: 10.1186/s12862-015-0375-4 (PMC4436027; doi:10.1186/s12862-015-0375-4)
Supplement: Additional file 3: Figure S3. — Analysis of sex chromosome multivalents of pachytene oocytes in Leptidea juvernica (a–d), L. sinapis (e–h) and L. reali (i–l) using FISH with the (TTAGG)n telomeric probe. Hybridization signals of the Cy3-dUTP-labelled telomeric probe (red) indicate chromosome ends. Chromosomes were counterstained with DAPI (blue). Figures (a–d), (e–h) and (i–l) show sex chromosome multivalents W1-nZ1-n: (a, e, i) merged images of the (TTAGG)n telomeric probe and DAPI staining; (b, f, j) DAPI images; note DAPI-highlighted heterochromatic segments of the W chromosomes; (c, g, k) hybridization pattern of the (TTAGG)n telomeric probe; (d, h, l) schematic drawings of the sex chromosome multivalents; yellow dots indicate the ends of individual chromosomes involved in the multivalents. Scale bar = 10 μm. [file 12862_2015_375_MOESM3_ESM.pdf]

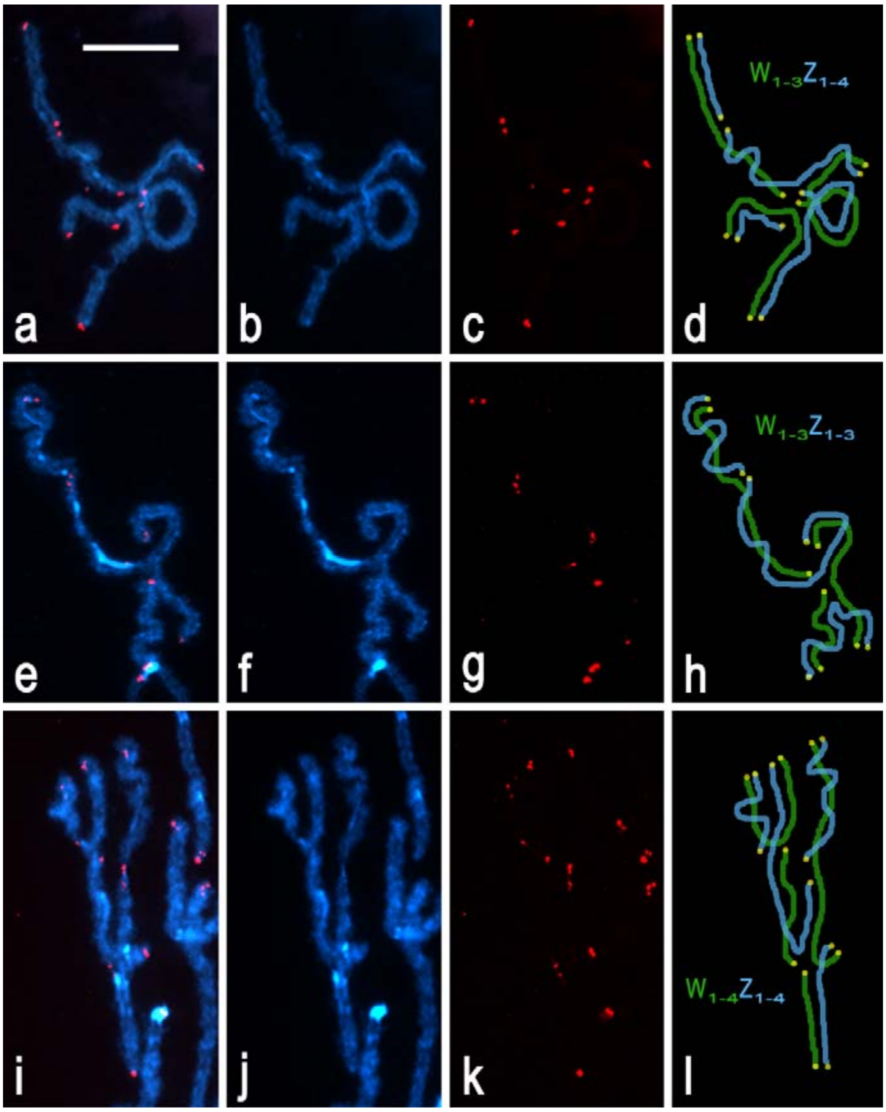

Figure S3 Analysis of sex chromosome multivalents of pachytene oocytes in *Leptidea juvernica* (a-d), *L. sinapis* (e-h), and *L. reali* (i-l) using FISH with the (TTAGG)$_n$ telomeric probe. Hybridization signals of the Cy3-dUTP-labelled telomeric probe (red) indicate chromosome ends. Chromosomes were counterstained with DAPI (blue). Figures (**a**-**d**), (**e**-**h**), and (**i**-**l**) show sex chromosome multivalents $W_{1\text{-}n}Z_{1\text{-}n}$: (**a**, **e**, **i**) merged images of the (TTAGG)$_n$ telomeric probe and DAPI staining; (**b**, **f**, **j**) DAPI images; note DAPI-highlighted heterochromatic segments of the W chromosomes; (**c**, **g**, **k**) hybridization pattern of the (TTAGG)$_n$ telomeric probe; (**d**, **h**, **l**) schematic drawings of the sex chromosome multivalents; yellow dots indicate the ends of individual chromosomes involved in the multivalents. Scale bar = 10 µm.
