# Supplementary material for: Dynamic karyotype evolution and unique sex determination systems in Leptidea wood white butterflies
Source: BMC Evol Biol. 2015 May 19;15:89. doi: 10.1186/s12862-015-0375-4 (PMC4436027; doi:10.1186/s12862-015-0375-4)
Supplement: Additional file 4: Figure S4. — Comparison of interphase nuclei sizes in three Leptidea species. The y-axis shows the number of pixels. Micrographs of interphase nuclei were taken from DAPI-stained spread preparations of wing discs from three different larvae of each Leptidea species, using the same resolution. In these micrographs, we measured the area of 144 nuclei of L. juvernica, 154 nuclei of L. reali and 130 nuclei of L. sinapis. The measurements were carried out using the software JMicroVision v1.2.7 [Roduit N: JMicroVision: Image analysis toolbox for measuring and quantifying components of high-definition images. Version 1.2.7. http://www.jmicrovision.com (accessed 27 March 2015)]. Calibration was performed using an image resolution so that the area of each nucleus was measured in pixels. The average size of nuclei was calculated for each species independently and then compared between species by one-way ANOVA using the software Statistica for Windows, version 8.0 (StatSoft, Inc., Tulsa, OK, USA). The comparison of interphase nuclei revealed no statistically significant between-species differences in their size (F (2, 9) = 0.6782; P = 0.5425). The mean (± S.E.) area of interphase nuclei was 22434 ± 2296 pixels for L. juvernica, 19781 ± 1965 pixels for L. reali and 19835 ± 1021 pixels for L. sinapis. [file 12862_2015_375_MOESM4_ESM.pdf]

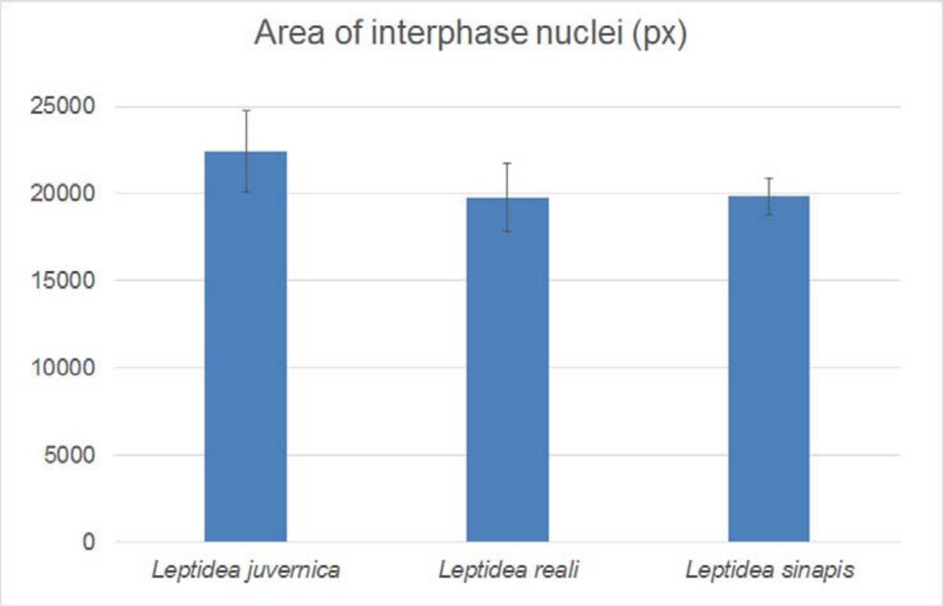

Figure S4 Comparison of interphase nuclei sizes in three *Leptidea* species. The y-axis shows the number of pixels. Micrographs of interphase nuclei were taken from DAPI-stained spread preparations of wing discs from three different larvae of each *Leptidea* species, using the same resolution. In these micrographs, we measured the area of 144 nuclei of *L. juvernica*, 154 nuclei of *L. reali*, and 130 nuclei of *L. sinapis*. The measurements were carried out using the software JMicroVision v1.2.7 [Roduit N: JMicroVision: Image analysis toolbox for measuring and quantifying components of high-definition images. Version 1.2.7. http://www.jmicrovision.com (accessed 27 March 2015)]. Calibration was performed using an image resolution so that the area of each nucleus was measured in pixels. The average size of nuclei was calculated for each species independently and then compared between species by one-way ANOVA using the software Statistica for Windows, version 8.0 (StatSoft, Inc., Tulsa, OK, USA). The comparison of interphase nuclei revealed no statistically significant between-species differences in their size ($F_{(2, 9)} = 0.6782$; $P = 0.5425$). The mean (± S.E.) area of interphase nuclei was 22434 ± 2296 pixels for *L. juvernica*, 19781 ± 1965 pixels for *L. reali*, and 19835 ± 1021 pixels for *L. sinapis*.
