# Supplementary material for: Dynamic karyotype evolution and unique sex determination systems in Leptidea wood white butterflies
Source: BMC Evol Biol. 2015 May 19;15:89. doi: 10.1186/s12862-015-0375-4 (PMC4436027; doi:10.1186/s12862-015-0375-4)
Supplement: Additional file 5: Table S1. — List of specimens included in phylogenetic analyses. Sequences obtained in this study are in blue, the other sequences were downloaded from GenBank and are representative for all the COI and ITS2 haplotypes of Leptidea sinapis, L. reali and L. juvernica identified in a previous study [41]. The haplotype numbers correspond to those in [41]. [file 12862_2015_375_MOESM5_ESM.pdf]

**Table S1 List of specimens included in phylogenetic analyses.** Sequences obtained in this study are in blue, the other sequences were downloaded from GenBank and are representative for all the *COI* and *ITS2* haplotypes of *Leptidea sinapis* , *L. reali* , and *L. juvernica* identified in a previous study [41]. The haplotype numbers correspond to those in [41].

| **Sample ID** | **Species** | ***COI* haplotype[1)]** | ***COI* accession number** | ***ITS2* haplotype[1)]** | ***ITS2* accession number** | **Locality** | **Country** |
|---|---|---|---|---|---|---|---|
| RVcoll.08-R368 | *L. juvernica* | **hj1** | KC865949 | **hj1** | KC865858 | Val di Tovo- Laghi | Italy |
| RVcoll.08-M322 | *L. juvernica* | **hj2** | HQ004596 | **hj1** | JF512789 | Valea Belchia, Harghita | Romania |
| RVcoll.10-A259 | *L. juvernica* | **hj6** | JF512651 | **hj1** | JF512783 | Gresse-en-Vercors, Isère | France |
| RVcoll.07-Z082 | *L. juvernica* | **hj7** | JF512648 | **hj1** | JF512768 | South Altai, Uspenka | Kazakhstan |
| RVcoll.09-X181 | *L. juvernica* | **hj9** | JF512715 | **hj2** | JF512788 | Kilternan, Dublin | Ireland |
| RVcoll.08-J396 | *L. reali* | **hr1** | JF512603 | **hr1** | JF512797 | Viladrau, Barcelona | Spain |
| RVcoll.03-H535 | *L. reali* | **hr6** | JF512617 | **hr1** | JF512790 | Saldes, Barcelona | Spain |
| RVcoll.07-E083 | *L. reali* | **hr7** | JF512712 | **hr1** | JF512795 | Roccaraso, L'Aquila | Italy |
| RVcoll.10-B385 | *L. sinapis* | **hs1** | JF512693 | **hs1** | JF512817 | Paril Village, Khadzhidimovo | Bulgaria |
| MF-63 | *L. sinapis* | **hs14** | KC866102 | **hs1** | KC865928 | Riala | Sweden |
| MF-174 | *L. sinapis* | **hs16** | KC866101 | **hs1** | KC865919 | Hodonín, South Moravia | Czech Republic |
| RVcoll.08-R436 | *L. sinapis* | **hs24** | GU675857 | **hs1** | KC865933 | Ames, Novais | Spain |
| RVcoll.10-C245 | *L. sinapis* | **hs5** | JF512697 | **hs1** | JF512847 | Příbram, Central Bohemia | Czech Republic |
| RVcoll.07-E237 | *L. sinapis* | **hs8** | JF512597 | **hs1** | JF512810 | Novalesa-Moncenisio, Torino | Italy |
| RVcoll.08-H720 | *L. sinapis* | **hs23** | KC865994 | **hs2** | KC865883 | Gairo | Sardinia |
| RVcoll.08-H769 | *L. sinapis* | **hs3** | KC866005 | **hs2** | KC865886 | Fozzaninco | Corsica |
| RVcoll.06-H637 | *L. sinapis* | **hs2** | JF513027 | **hs3** | KC865931 | Landman, Zyryanovsk | Kazakhstan |
| RVcoll.06-K558 | *L. sinapis* | **hs1** | JF513036 | **hs4** | KC865939 | Bădeni, Cluj | Romania |
| RVcoll.10-C251 | *L. sinapis* | **hs1** | JF512699 | **hs5** | JF512832 | Monte di Malo, Veneto | Italy |
| MF-F12 | *L. juvernica* | **hj10** | KC866126 | | | Riala | Sweden |
| GenBank | *L. juvernica* | **hj11** | EF599645 | | | Barje | Slovenia |

| | | | | | | | |
|---|---|---|---|---|---|---|---|
| GenBank | *L. juvernica* | **hj12** | EF599643 | | | Barje | Slovenia |
| GenBank | *L. juvernica* | **hj13** | EF599640 | | | Vrhnika | Slovenia |
| RVcoll.08-Y008 | *L. juvernica* | **hj3** | JF512578 | | | Peterhof, St. Petersburg | Russia |
| RVcoll.11-G200 | *L. juvernica* | **hj4** | KC865980 | | | Narymski Mts. | Kazakhstan |
| RVcoll.11-G219 | *L. juvernica* | **hj5** | KC865982 | | | Balgyn | Kazakhstan |
| GenBank | *L. juvernica* | **hj8** | GU655014 | | | Neustadt/Donau, Plattenberg, Bavaria | Germany |
| RVcoll.09-X183 | *L. juvernica* | **hj9** | JF512716 | | | Gortmore Point, Lough Derg, Tipperary | Ireland |
| RVcoll.10-C269 | *L. reali* | **hr2** | JF512616 | | | Cascia, Perugia | Italy |
| RVcoll.10-C270 | *L. reali* | **hr3** | JF512704 | | | Sibillini Mountains | Italy |
| RVcoll.08-H468 | *L. reali* | **hr4** | GU676645 | | | Hormiguera, Cantabria | Spain |
| MF-90 | *L. reali* | **hr5** | KC866117 | | | Pla de la Calma, Montseny | Spain |
| RVcoll.07-C210 | *L. sinapis* | **hs10** | JF512592 | | | Schitul Pahomie, Vâlcea | Romania |
| RVcoll.06-H631 | *L. sinapis* | **hs11** | JF513025 | | | Landman, Zyryanovsk | Kazakhstan |
| RVcoll.06-H632 | *L. sinapis* | **hs12** | JF513047 | | | Landman, Zyryanovsk | Kazakhstan |
| GenBank | *L. sinapis* | **hs13** | HM393183 | | | Zahmer Kaiser, Aschinger Alm, Tyrol | Austria |
| RVcoll.10-C262 | *L. sinapis* | **hs15** | KC866089 | | | Corciano, Perugia | Italy |
| MF-155 | *L. sinapis* | **hs17** | KC866100 | | | Sant Celoni, Barcelona | Spain |
| RVcoll.11-K071 | *L. sinapis* | **hs18** | KC866098 | | | Mala Reka, Mavrovo | Macedonia |
| GenBank | *L. sinapis* | **hs19** | GU688515 | | | Ruhpolding, Bavaria | Germany |
| RVcoll.10-B320 | *L. sinapis* | **hs20** | KC866088 | | | Studen Kladenets, Krumovgrad | Bulgaria |
| GenBank | *L. sinapis* | **hs21** | GU688533 | | | Lenggries Isarauen, Bavaria | Germany |
| RVcoll.11-K078 | *L. sinapis* | **hs22** | KC866104 | | | Mala Reka, Mavrovo | Macedonia |
| RVcoll.11-J544 | *L. sinapis* | **hs25** | KC866097 | | | Manosque, Alpes-de-Haute-Provence | France |
| RVcoll.07-Z211 | *L. sinapis* | **hs4** | JF513046 | | | Saur Mts., Malyi Zhemeney | Kazakhstan |
| RVcoll.07-E250 | *L. sinapis* | **hs6** | JF513034 | | | NE Bézaudun-sur-Bine, Drôme | France |
| RVcoll.09-X562 | *L. sinapis* | **hs7** | KC866082 | | | Sorauren, Navarra | Spain |
| RVcoll.07-D938 | *L. sinapis* | **hs9** | JF513026 | | | Ciupercenii de Olteț, Gorj | Romania |

| | | | | | | | |
|---|---|---|---|---|---|---|---|
| Nz091 | *L. amurensis* | | JF512621 | | | Bulgan | Mongolia |
| RVcoll.10-C186 | *L. amurensis* | | JF512622 | | JF512841 | Jiexiu county, Shanxi | China |
| RVcoll.09-V207 | *L. duponcheli* | | JF512569 | | JF512852 | Oraison, Alpes de Haute Provence | France |
| MF-1 | *L. duponcheli* | | KC866120 | | | Skopje | Macedonia |
| RVcoll.10-C189 | *L. lactea* | | JF512717 | | JF512849 | Qin Ling Shan, Madao, Liuba County, Shaanxi | China |
| RVcoll.10-C195 | *L. lactea* | | JF512718 | | | Qin Ling Shan, Zhouzhi (Erqu) County, Shaanxi | China |
| RVcoll.07-Z124 | *L. morsei* | | JF512618 | | | South Altai, Markakol | Kazakhstan |
| RVcoll.08-M498 | *L. morsei* | | HQ004591 | | JF512840 | Bădeni, Cluj | Romania |
| Ls_LF04 | *L. sinapis* | | KM488575 | | KM488591 | NP Podyjí, South Moravia | Czech Republic |
| Ls_LF11 | *L. sinapis* | | KM488578 | | KM488594 | NP Podyjí, South Moravia | Czech Republic |
| Ls_LF12 | *L. sinapis* | | KM488579 | | KM488595 | NP Podyjí, South Moravia | Czech Republic |
| Ls_LF14 | *L. sinapis* | | KM488580 | | KM488596 | NP Podyjí, South Moravia | Czech Republic |
| Ls_LF15 | *L. sinapis* | | KM488581 | | KM488597 | NP Podyjí, South Moravia | Czech Republic |
| Ls_LF16 | *L. sinapis* | | KM488582 | | KM488598 | NP Podyjí, South Moravia | Czech Republic |
| Lr_LF21 | *L. reali* | | KM488584 | | KM488600 | NP Montseny, Barcelona | Spain |
| Lr_LF22 | *L. reali* | | KM488585 | | KM488601 | NP Montseny, Barcelona | Spain |
| Lr_LF24 | *L. reali* | | KM488586 | | KM488602 | NP Montseny, Barcelona | Spain |
| Lr_LF25 | *L. reali* | | KM488587 | | KM488603 | NP Montseny, Barcelona | Spain |
| Lr_LF61 | *L. reali* | | KM488588 | | KM488604 | NP Montseny, Barcelona | Spain |
| Lr_LF62 | *L. reali* | | KM488589 | | KM488605 | NP Montseny, Barcelona | Spain |
| Lj_LF06 | *L. juvernica* | | KM488576 | | KM488592 | České Budějovice, South Bohemia | Czech Republic |
| Lj_LF07 | *L. juvernica* | | KM488577 | | KM488593 | České Budějovice, South Bohemia | Czech Republic |
| Lj_LF18 | *L. juvernica* | | KM488583 | | KM488599 | České Budějovice, South Bohemia | Czech Republic |
| Lj_LF67 | *L. juvernica* | | KM488590 | | KM488606 | České Budějovice, South Bohemia | Czech Republic |
